# Design, Fabrication, Structure Optimization and Pressure Sensing Demonstration of COC Piezoelectret Sensor and Sensor Array

**DOI:** 10.3390/mi13081177

**Published:** 2022-07-26

**Authors:** Hui Wang, Xiaolin Wang, Matthew Wadsworth, Mohammad Faisal Ahmed, Zhe Liu, Changchun Zeng

**Affiliations:** Department of Industrial and Manufacturing, High-Performance Materials Institute, FAMU-FSU College of Engineering, Florida State University, Tallahassee, FL 32310, USA; hw11b@eng.famu.fsu.edu (H.W.); xw15d@fsu.edu (X.W.); msw17c@fsu.edu (M.W.); mfa13@fsu.edu (M.F.A.); zl15c@fsu.edu (Z.L.)

**Keywords:** COC piezoelectrets, piezoelectric coefficient, response surface method, pressure mapping

## Abstract

This study reported on the design and fabrication of a pseudo-piezoelectric material (piezoelectret) from cyclic olefin copolymer (COC) based on a micropillar structure. The fabrication feasibility of such structure was explored and piezoelectret with the good piezoelectric activity (characterized by quasi-static piezoelectric coefficient *d*_33_) was demonstrated. Response surface method with a central composite design was employed to investigate the effects of the structure parameter on the piezoelectric coefficient *d*_33_. An optimal structure design was obtained and was validated by experiments. With the optimal design, *d*_33_ can reach an exceptional high value of ~9000 pC/N under low pressure. The charging process and the electrical and electromechanical characteristics were further investigated by experimentation and modeling. We further demonstrated the scalability of the fabrication process and demonstrated the application of these sensors in position specific pressure sensing (pressure mapping).

## 1. Introduction

Piezoelectric material can convert mechanical energy to electrical energy. Currently, the most commonly used piezoelectric material is ceramic based piezoelectric materials. Porous polymer piezoelectric materials (piezoelectric foams or piezoelectrets) were first investigated in Finland in 1989 [1]. The piezoelectric properties arise from the macroscopic dipoles inside the material [2,3]. Compared to traditional piezoelectric material, polymer piezoelectrets are flexible, non-toxic, lightweight and more affordable. Their piezoelectric activity was comparable to or better than piezoelectric ceramics. A variety of polymers, including polypropylene (PP) [4,5], cyclic olefin copolymer (COC) [6,7] and fluorinated ethylene propylene (FEP) [8,9], were used in the fabrication of piezoelectrets.

Piezoelectricity of the piezoelectrets results from the change of the macro-level dipole moment induced by the mechanical deformation of the material under stress. To achieve high performance piezoelectrets, structures with large deformability were highly desired. Various structures, including lens-shape structure [3], non-overlap structures [6,7] and other structures [8,9], are successfully fabricated in predominantly two approaches: (i) direct foaming of polymer by using a gas to expand and generate porosity in the polymer and (ii) generation of pre-machined cavitation in the structure units followed by assembly/fusion of the units [10,11,12,13,14]. For the first approach, it is difficult to obtain the well-defined structures as the direct foaming process possesses stochastic characteristics, which are nearly impossible to control in the manufacturing process. For the second approach, porosity and the pore distribution were better controlled; however, the dimension accuracy was still significantly affected in the bonding area due to the introduction of the high temperature. New piezoelectret structures that can realize high piezoelectric activity and their fabrication approaches are in high demand for the advancement of this type of smart materials.

In this study, a micropillar-based structure was explored for piezoelectret fabrication. Such an approach potentially addresses the shortcomings of other existing technology previously discussed. First, the approach allows for the design and fabrication of structures with well-defined parameters. In addition, the printing process examined in this study is significantly simpler and involves fewer steps. This not only improves the technology efficiency but also reduces the processing time and improves structural fidelity, the major difficulties for the pre-machining/fusion method.

**Figure 1:**
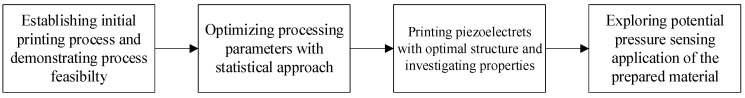
Overview of micropillar-based piezoelectret design fabrication and application.

Figure 1 shows the overall design of the current study. The feasibility studies of the proposed approach were conducted first. Using an initial design, the 3D printing fabrication process was established and the piezoelectric sensitivity of the fabricated piezoelectret was demonstrated. A statistical approach of response surface method was used to optimize the structure parameters for maximum piezoelectric activity. Subsequently, piezoelectrets with optimized structures were fabricated and their electrical and electromechanical behaviors were investigated. Finally, this study demonstrated the pressure sensing capabilities of the prepared piezoelectret.

## 2. Materials and Experimental Details

### 2.1. Materials and Fabrication Equipment

The base polymer used for the fabrication was a cyclic olefin copolymer (COC). COC is a copolymer of ethylene and a cyclic comonomer (typically norbornene or tetracyclododecene). COCs demonstrate excellent processability, environmental stability, low dielectric constant, low dielectric losses and excellent mechanical properties and, particularly, good thermal stability [15]. Several studies had been conducted regarding the piezoelectric foams for this type of material [16,17,18,19].

The COC film used in this study was grade 6017 TOPAS from Advanced Polymers (Florence, KY, USA). The COC film was 50.8 µm thick. The epoxy resin for deposition was SU-8 2002 from Micro Chem Company. A needle was used for depositing epoxy resin onto the COC film. The deposition system was assembled with a tabletop 3D printer (Zen Toolworks, ZEN7123D, Concord, CA, USA), a syringe and a pump.

### 2.2. Fabrication of COC Piezoelectret

**Figure 2:**
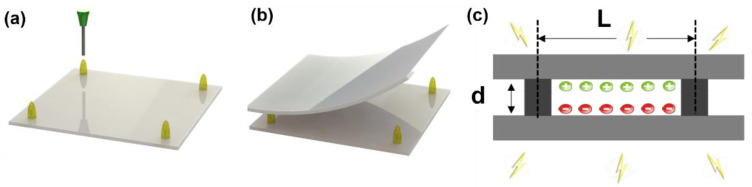
Schematic of micropillar based piezoelectret fabrication (**a**). Deposition of SU-8 epoxy resin by 3D printing machine in xyz-axis (**b**). Assembly of the micropillar structure with COC film by UV-curing the SU-8 epoxy resin (**c**). The 2D schematic view of the cross section of prepared piezoelectret with artificial charge voids inside. The micropillar structure were rendered piezoelectric by charging.

Piezoelectrets are materials with cavity/voids. Charges of opposite signs may be deposited on the top and bottom surfaces of the voids to form macro-dipoles, which results in piezoelectricity, as shown in Figure 2c. In this work, 3D printing was used to generate the void structures. The 3D printing enables precise control of the dimensions of the void structure, which offers better control and optimization of the piezoelectric activity and allows for the fabrication of sensor arrays. Figure 2c shows the basic cell of the designed structure. Two main design parameters in the fabrication process are the span between epoxy resin pillar (L) and the pillar height (d). They have convoluted effects on the piezoelectret’s elastic modulus. The pillar height will affect the charging process, which will affect the piezoelectret’s performance. In the previous work of fabricating the porous piezoelectret using foaming process, such parameters were difficult to precisely control. A custom-built 3D printing machine with xyz stage was used with SU-8 epoxy resin and COC film. The UV cure epoxy resin can locally bond the COC films, which enables the piezoelectret with precise dimension without having large deformations compared to the piezoelectrets fabricated with fusion bonding process.

The overall structure consisted of two COC films separated by epoxy pillar material. The preparation procedures of piezoelectret can be divided into three general steps. First, micro-pillars were fabricated by SU-8 UV curing epoxy resin with the help of the 3D printing based deposition system. Second, the SU-8 epoxy resin was cured under UV light and the polymer film was bonded together. Finally, contact charging was applied to obtain piezoelectricity for the material. The detailed fabrication process was illustrated in Figure 2.

In the first step, micropillar was deposited onto the four corners of COC film by a 3D printing based deposition system. The deposition system consisted of an xyz-axis 3D printer controlled by a computer, a syringe pump controlled by syringe infusion pump (Cole-Parmer dual-syringe infusion pump, EW-74900-10) and a needle 0.3 mm in diameter. The position of the needle could be precisely controlled at the xyz stages. The flow rate was controlled using the syringe infusion pump and was kept constant for the different experiments. The height of the micro-pillar was controlled by differing the deposition time. Different pillar heights and pillar–pillar distances were chosen to study the material sensitivity corresponding to different design parameters.

In the second step, the top layer of COC film was bonded with the micro-pillars. The bottom COC layer was heated to 110 °C to slightly soften the SU-8 epoxy resin. Top layer of COC film was then applied onto the micro-pillars and pressure was applied by a glass plate. The sample was then put under UV light (Dymax light curing system, Model 5000 Flood) for 30 s to cure the resin. The use of the resin deposition followed by curing allowed for more accurate control of the structure dimensions.

In the third step, contact charging was used to charge the structure. The sample was sputter coated with metal electrodes on both sides of the material. The material then underwent a contact charging with high voltages using a Heinzinger PNC 1000-6 ump.

### 2.3. Characterization of COC Piezoelectret

#### 2.3.1. Quasi-Static Piezoelectric Coefficient

Quasi-static piezoelectric coefficient is a common parameter for measuring the performance of piezoelectric material, especially piezoelectrets [3], which was determined by:

(1) $$d_{33}=\frac{Q}{F}=\frac{\sigma }{p}$$

where *d*_33_ is quasi-static piezoelectric coefficient, *Q* is amount of charge, *F* is applied force, *σ* is charge density, *p* is applied pressure. A preload of 1.96 kPa was applied to the sample before quasi-static piezoelectric coefficient measurements to eliminate the air gap effect ^4^. Pressures from 0.49 kPa to 4.9 kPa were then applied onto the material to measure the piezoelectric coefficient. In the experiments, the induced charge was measured by an electrometer (Keithley 6517A).

#### 2.3.2. Electric Hysteresis Loop

To investigate the charge build up process in the air gaps of samples with optimized structures, hysteresis loop measurement were conducted using Precision Premier II (RADIANT) connected to a high voltage interface. Electrodes were sputter coated over a 10 mm × 10 mm area. Bipolar charging voltages from 500 V to 8000 V were applied on the sample to measure the polarization of the sample.

### 2.4. Pressure Sensing System Fabrication and Development

**Figure 3:**
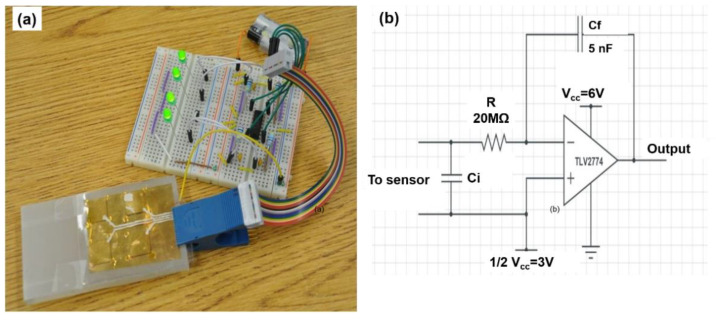
(**a**) Photo of a fabricated 2 × 2 micropillar-based COC piezoelectret sensor array with application for pressure mapping; (**b**) Piezoelectret pressure sensing circuit.

To demonstrate the scalability of the fabrication process and the device performance, a 2 × 2 piezoelectric sensor array was fabricated, and their pressure sensing capability demonstrated. Fabrication of the sensor array fabrication was similar to that of the single sensor. Epoxy pillars for multiple sensor unit cells were deposited on the COC film, and then the top and bottom COC films were bonded together when the UV epoxy resin was cured. Figure 3a shows the prototype system consisting of the sensor array and the associated electronic circuits (Figure 3b). Sensors were connected to a 4 channel CMOS operational amplifier to amplify the signal. The output signal of piezoelectric sensor is a high impedance signal that cannot be directly measured by a multimeter. A charge amplifier (MOSFET, TLV2774 from Texas Instrument, Dallas, TX, USA) was used to convert the signal to low impedance signal. The charge amplifier circuit was designed to work in the voltage mode since this mode can reduce the negative effects of the capacitance on the measurements of signals of the connection cables in the circuit. The charge signal was connected to the gate side of the amplifier and the output signal was a voltage signal. A power supply of 6 V was applied, and bias voltage was set to be 3 V. The output voltage was read by a multimeter (Fluke 179). The LED lights were used as an indicator for presence/absence (“on”/“off”) of pressure.

## 3. Result and Discussion

### 3.1. Piezoelectric Activity of COC Piezoelectret: Feasibility

**Figure 4:**
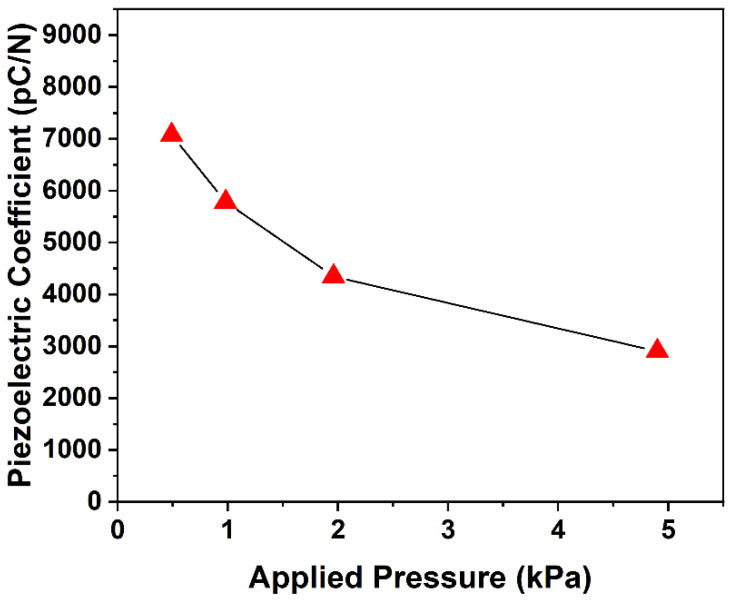
Quasi-static piezoelectric coefficient of the printing COC piezoelectret (L = 15 mm, h = 1 mm); charging voltage was 5 kV.

A COC piezoelectret (L = 15 mm, h = 1 mm) was fabricated to test the feasibility of the 3D printing approach. Figure 4 shows the quasi-static $d_{33}$ piezoelectric coefficient of piezoelectret. The $d_{33}$ can reach approximately 7000 pC/N at an applied pressure of 0.49 kPa, which is an extremely high value compared to the sensitivity of other materials [7,16,17,18,19]. Although the piezoelectric coefficient decreased with increasing pressure, it remained high in the range of testing pressure, reaching 3500 pC/N at 4.9 kPa. The results clearly indicate that 3D printing highly sensitive piezoelectrets with the designed structure is feasible.

### 3.2. Statistical Modeling and Experimental Validation of Optimized Structure for Maximized Piezoelectric Activity

The quasi-static piezoelectric coefficient $d_{33}$ is largely dictated by two factors [20,21]: $d_{33}\propto \sigma /Y$, where $d_{33}$ is piezoelectric coefficient, σ is charge density inside the porous structure (artificial void), *Y* is elastic modulus. For the structures fabricated, both the charge density and the elastic modulus are influenced in a complicated manner by the two design parameters: the height of the micro-pillar (d) and the distance between pillars (L), as shown in Figure 2c. The charging of the piezoelectret is by dielectric barrier discharge for which the threshold break down voltage follows the Paschen law (Equation (2)) [22], after which the charge density increases linearly until back charging occurs at excessively high voltage. To achieve a higher piezoelectric coefficient $d_{33}$, a lower threshold breakdown voltage is desired, which would result in higher charge density at the same charging voltage.

(2) $$V_{bd}=\frac{apd_{gas}}{\ln(pd_{gas})+b}$$

The breakdown voltage *V_bd_* is a highly nonlinear function of both various materials related parameters or constants (*a*, *b*, *P*), and the air gap thickness *d_g_*, which is closely related to the pillar height d.

The span between pillars (L) primarily affects the elastic modulus *Y* and the stability of the structure. In general, a smaller span would result in a higher elastic modulus and lower piezoelectric activity. On the other hand, at an exceedingly high L (or L/d), the structure may collapse because of the insufficient structure rigidity, leading to the loss the piezoelectric sensitivity. The sinking of the center of the top COC film may also occur at high L, leading to a reduction in the air gap thickness and affecting charging and the piezoelectric coefficient of the COC piezoelectrets.

Because of the complicated and convoluted relationship between the piezoelectric activity and the structure parameters, modeling the structure–property relationship for optimal piezoelectric performance was challenging. Existing models [20,21,23,24,25], based on different geometric structures and simplifications do not suit the structures in this study and are incapable of accurate predictions for optimized structure design.

On the other hand, despite lacking physical meaning, statistical models based on experimental data can provide excellent predictions so long as the new data the predicted data are in the proximity of existing experimental data [26,27]. Because of the complicated relationships between pillar–pillar distance, micro-pillar height and piezoelectric coefficient, instead of using analytical approach, a statistical approach is better suited to determine the optimal for such structure parameters. Considering the curvature in the system, a statistical approach of response surface method (RSM) was used as a regression method to build the statistical models [28], with the objective to optimize a response (piezoelectric coefficient *d*_33_) that is influenced by several independent variables (d and L). The method estimates the function between the response and input variable, which is a convex or concave function for most of the case. Subsequently, a gradient decent algorithm is generally used to find the possible maximum or minimum value of the function. Design Expert software was used to produce the response surface. The prediction would then be validated by experiments. To establish the model, a series sets of inputs and responses from experiments would be required to generate reliable statistical models and inferences. A central composite factorial (CCD) design of experiment was utilized to minimize the number of needed experiments.

**Figure 5:**
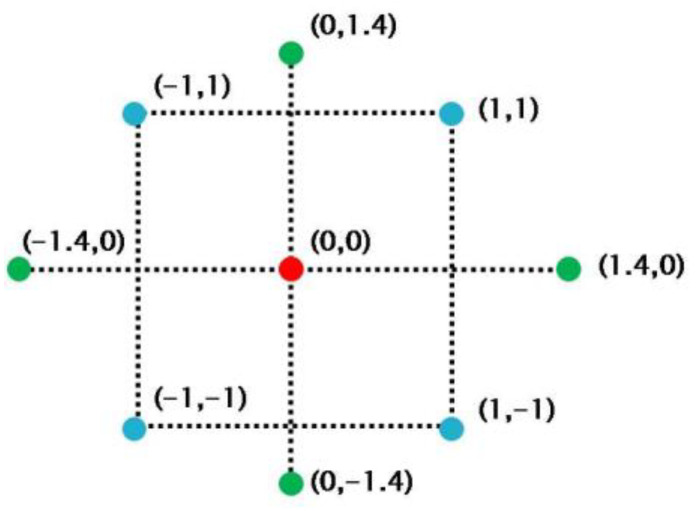
Central composite design to optimize pillar–pillar distance and pillar height for maximized piezoelectric activity.

Figure 5 shows the scaled factors used in the central composite design. For each set of parameters, three replicates were conducted. In this set of experiments, a fixed charging voltage of 5 kV was used. Table 1 lists the values of structure design variables in all experiments. The range of each variable was determined by the preliminary estimates and calculation results. The pillar–pillar distance was chosen from 11 mm to 19 mm. The range of pillar height was 0.3~1.8 mm. A total number of 27 experiments were conducted with each parameter condition replicated 3 times.

**Figure 6:**
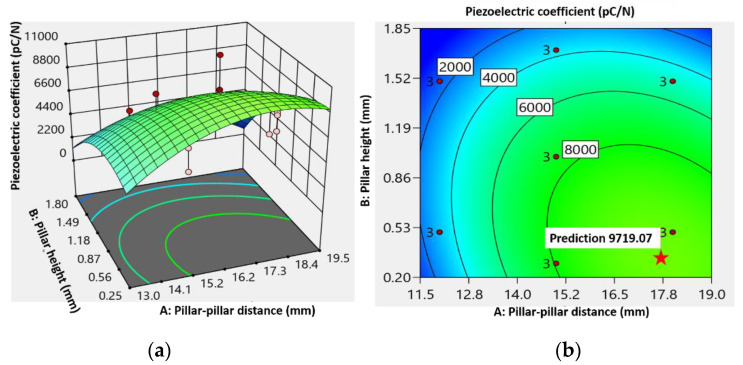
(**a**) Response surface of piezoelectric coefficient corresponding to piezoelectrets with different structure parameters. Red dots in the figure were experimental measurements, and the green surface was the model predicted response surface. (**b**) The 2D projection of the response surface showing the maximized $d_{33}$ and the associated optimal structure parameters (pillar height and pillar–pillar distance). This optimum is indicated by the star on the figure.

Figure 6a shows the response surface of piezoelectric coefficient obtained from the experiments. Figure 6b is a 2D projection of the response surface. The red symbols showed the experimental value. In the relationship between piezoelectric coefficient ($d_{33}$) and pillar height (d), pillar–pillar distance (L) is generally a convex function. Both pillar height and pillar–pillar distance were significant (*p* = 0.0005, 0.0172, respectively) in affecting the piezoelectric coefficient. The optimal pillar height was 0.33 mm and pillar–pillar distance was 17.7 mm. The maximum piezoelectric coefficient was predicted to be 9719 pC/N (95% Confidence, 6084–13,352 pC/N) by the statistical model. The large range may be due to the pure statistical nature of the model, which omitted details and complex coupled electromechanical properties of the materials.

Following the model prediction, COC piezoelectrets were fabricated using the optimal structure parameters d = 0.3 mm and L = 18 mm. The measured $d_{33}$ was ~9000 pC/N. The comparable value between the model prediction and the experimental measurements appears to verify the validity of the statistical model.

### 3.3. Electrical and Piezoelectric Properties of COC Piezoelectrets with the Optimized Structure

We studied the charging behavior of COC piezoelectrets with the optimal design. Although generally incapable of locating the optimal conditions for maximized piezoelectric coefficient, physics-based engineering models ^22^ may be used to calculate the piezoelectric coefficients with provided structure parameters and facilitate the understanding of the governing physical phenomena. We thus modelled the piezoelectric $d_{33}$ of the piezoelectret of the optimal design using a physics-based approach and compared the model prediction with that from the statistical model and the experimental value.

#### 3.3.1. Hysteresis Loop

**Figure 7:**
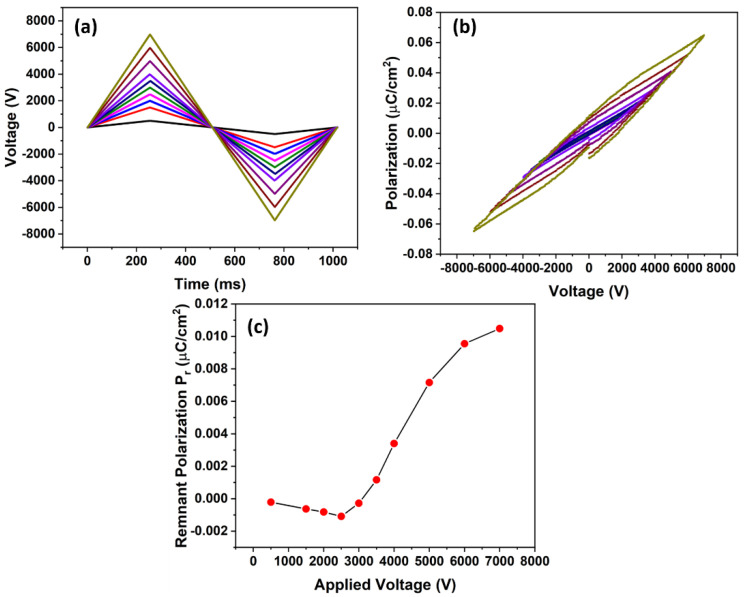
(**a**) Applied voltage over time on the sample (**b**) Hysteresis loop of the prepared sample (**c**) Quasi-permanent charge build up in the micropillar-based COC piezoelectret.

The charge-build up process for the piezoelectrets was characterized by the hysteresis loop [29,30,31]. Figure 7a shows the applied voltage on the surface of the sample over time during the electric hysteresis loop measurements. Bipolar voltages were applied to the electrodes of the sample at a cycle time of 1 s. Figure 7b shows the hysteresis loops of the sample. From the hysteresis loop, the quasi-permanent polarization can be extracted, which is shown in Figure 7c. The dipole inside the air gap started to build up with an applied voltage of approximately 2500 V. Below this threshold voltage, nearly no charge accumulated during the charging process. When the applied voltage was higher than the threshold breakdown voltage, dielectric barrier discharge occurred and the charge started to build up in the artificial void. In particular, the quasi-permanent charge was approximately linear proportional to the applied voltage after the applied voltage reached the threshold breakdown voltage, which indicates that the piezoelectric coefficient can be further increased by increasing the contact charging voltage.

To understand quantitatively this charging threshold voltage, a simplified model was derived below based on a previous study [20,21].

**Figure 8:**
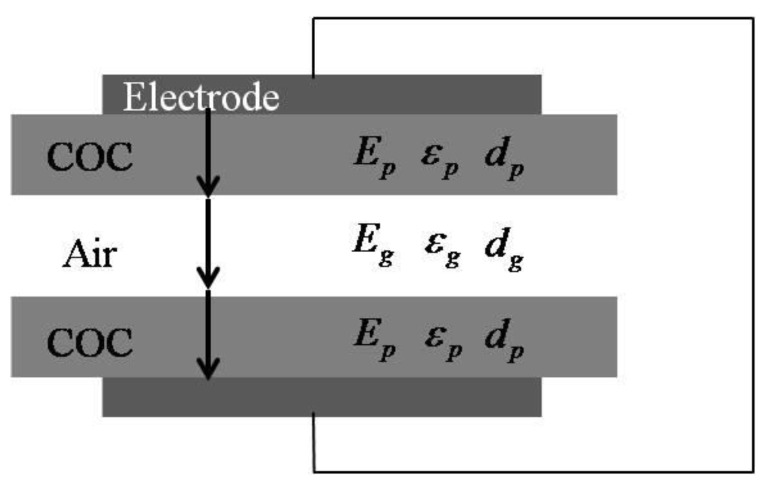
Simplified model for calculating breakdown voltage.

Figure 8 shows the schematics of the fabricated structure consisting of two layers of the COC polymer and an air gap in between. The electric field was built up during the contact charging. According to Gauss’ theorem and Kirchhoff’s second law

(3) $$\varepsilon_{0}\varepsilon_{p}E_{p}=\varepsilon_{0}E_{g}$$

(4) $$2d_{p}E_{p}+d_{g}E_{g}=V_{total}$$

where *ε***_0_** is dielectric permittivity of vacuum, *ε_p_* is relative permittivity of COC, *d_g_* and *d_p_* are thickness of air and COC film, respectively; *E_g_* and *E_p_* are electric field strength in air and COC, respectively; and *V_total_* is the applied voltage. The discharge inside the air gap was dielectric barrier discharge, which follows the Paschen law, for which the critical breakdown electric field strength is determined by:

(5) $$E_{bd}=\frac{ap}{\ln(pd)+b}$$

where *E_bd_* is the breakdown electric field strength, a is 4.36 × 10^7^ V/(atm·m), *P* is pressure (1 atm in the study), d is the thickness of gas (0.3 mm in this study), *b* equals to 12.8. From above, the critical breakdown voltage can be determined by:

(6) $$V_{bd}=\frac{ap}{\ln(pd_{g})+b}(\frac{2d_{p}}{\varepsilon_{p}}+d_{g})$$

For this study, with an air gap thickness of 0.3 mm, the breakdown voltage was calculated to be 3190 V. The model prediction from Equation (6) agreed relatively well with experimental measurement (~2500 V). The higher model predicted threshold breakdown voltage can be understood by the deflection of the sample structure; therefore, reduction in the air gap thickness during the bonding process was taken into consideration. According to Equation (6), the breakdown threshold voltage would decrease with decreasing air gap thickness *d_g_*.

#### 3.3.2. Physics Based Model on Piezoelectric Coefficient

Using the experimental measured charge density and previously developed layer model, we modelled the *d*_33_ of the material. When measuring the quasi-static piezoelectric coefficient, samples were applied with pressure *σ_stress_* and induced charge was accumulated on the electrodes. By Gauss theorem and Kirchhoff law:

(7) $$\varepsilon_{0}\varepsilon_{p}E_{p}=\sigma_{in}$$

(8) $$\varepsilon_{0}\varepsilon_{g}E_{g}-\varepsilon_{0}\varepsilon_{p}E_{p}=\sigma$$

(9) $$2d_{p}E_{p}+d_{g}E_{g}=0$$

where *ε***_0_** is the dielectric permittivity of vacuum, *ε_p_* is the relative permittivity of COC, *ε_g_* is the relative permittivity of air, *σ_in_* is induced charge, *σ* is surface charge density inside the air gap and *E_p_* and *E_g_* are the electric field strength in COC and air, respectively.

From Equations (7)–(9), the induced charge can be calculated as:

(10) $$\sigma_{in}=-\frac{\varepsilon_{0}\varepsilon_{p}d_{g}\sigma }{2d_{p}\varepsilon_{0}\varepsilon_{g}+d_{g}\varepsilon_{0}\varepsilon_{p}}$$

The quasi-static piezoelectric coefficient can then be expressed as:

(11) $$\begin{array}{l} d_{33}=\frac{\Delta \sigma_{in}}{\Delta \sigma_{stress}}=\frac{\frac{\partial \sigma_{in}}{\partial d_{g}}}{\frac{\Delta \sigma_{stress}}{\Delta d_{g}}}=\frac{-\frac{\varepsilon_{0}\varepsilon_{p}2d_{p}\varepsilon_{0}\varepsilon_{g}\sigma }{{\left(2d_{p}\varepsilon_{0}\varepsilon_{g}+d_{g}\varepsilon_{0}\varepsilon_{p}\right)}^{2}}}{\frac{Y_{f}}{2d_{p}+d_{g}}}\\ =-\frac{\varepsilon_{p}\varepsilon_{g}2d_{p}\left(2d_{p}+d_{g}\right)}{{\left(2d_{p}\varepsilon_{g}+d_{g}\varepsilon_{p}\right)}^{2}}\cdot \frac{\sigma }{Y} \end{array}$$

Equation (11) yields the quantitative prediction of the piezoelectric coefficient $d_{33}$ as a function of the geometric parameters (*d_g_* and *d_p_*), the dielectric properties (*ε_p_* and *ε_g_*), and the electrical (*σ*) and mechanical (*Y*) properties of the piezoelectret. However, to calculate the piezoelectric coefficient, the values of the surface charge density σ and the elastic modulus of the piezoelectret are required. The surface charge density σ can be obtained directly from the hysteresis loop measurement, which was 0.00716 μC/cm^2^ for this study. Calculating the elastic modulus of the micropillar-based structure is more complicated. Under compressive stress, the deformation is not uniform and is position/location dependent. A methodology is needed to estimate an “apparent strain,” and to establish the stress–strain relationship from which the “apparent modulus” can be deduced. Herein, we utilized finite element analysis to achieve this using COMSOL Multiphysics software (Version 5.2a, COMSOL Inc., Burlington, MA, USA).

**Figure 9:**
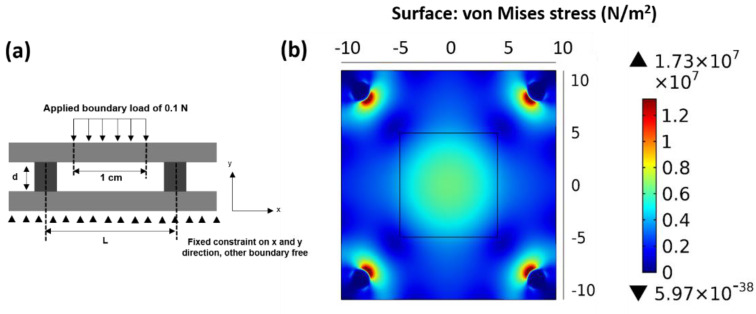
(**a**) Unit structure and boundary conditions for the finite element analysis (FEA) on the micropillar-based structure; (**b**) A typical FEA results from stress distribution for the calculation of apparent elastic modulus of the micropillar-based structure, L = 18 mm.

Figure 9a shows the geometry for the simulation. The piezoelectret was represented by two layers of solid polymer and an air gap separated by the micropillars with a height of d and span of L. In the simulation, a small force of 0.1 N (to ensure linear elasticity) was applied uniformly on the electrode indicated by the center area in Figure 9a,b. The apparent strain was determined by averaging the deflection over the electrode area. The apparent modulus was determined from the stress and apparent strain. Simulations suggest that the apparent elastic modulus was 0.63 kPa for the piezoelectret with L = 18 mm.

With the surface charge density and elastic modulus values, the piezoelectric coefficient was calculated using Equation (11) to be 15,476 pC/N. A piezoelectret with the same pillar distance and height was fabricated and the piezoelectric coefficient measured. The model-predicted value was about 70% higher than the experimentally measured value of 9000 pC/N. Such a difference may result from the simplifications assumed in the establishment of the model, as well as in the calculation of the apparent elastic modulus. Nevertheless, qualitatively the model is in agreement with the experiment. The physical-based model indeed provided the insights that the main origin of the high piezoelectric activity is from the low elastic modulus of the structure.

### 3.4. Pressure Sensing Application of the Prepared Material

We demonstrated the piezoelectret’s potential pressure sensing capability with a pressure sensing prototype. The system consisted of a sensor array of four sensors in 2 × 2 configuration and the associated electronic circuits. Each of the four LED lights was connected to one sensor output, and the battery power supply. The sensing circuit was designed such that the voltage output in regard to the input charge amount followed Equation (12).

(12) $$V_{o}=-\frac{q}{c_{f}}+\frac{V_{cc}}{2}$$

where *V_o_* is total output voltage supplied to the LED light, *q* is the input charge induced from pressure applied to the piezoelectrets, *C_f_* is feedback capacitance, *V_cc_* is power supply voltage. When no pressure was applied on the sensor, the output voltage would be half of the power supply voltage, sufficient to power on the LED light. When pressure is applied, the output voltage will decrease and turn off the LED light at certain pressure.

**Figure 10:**
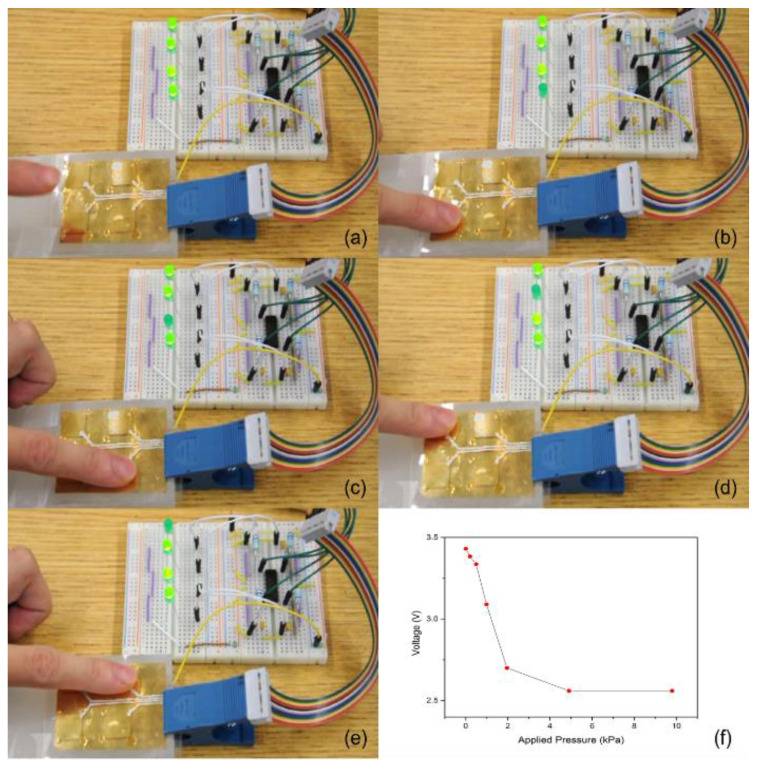
(**a**–**e**) Pressure sensing result (**f**) Pressure–voltage relationship of the testing piezoelectret.

Figure 10a shows the sensing results without applying pressure—all four LED lights were powered on. When pressure was applied to each of the four piezoelectret sensors, the corresponding LED light was turned off (Figure 10b–e), demonstrating successful pressure sensing. Sensors remained functional when repeatedly hard pressed more than 100 cycles during which the top and bottom COC films were in direct contact. This indicates that the charge was deeply trapped inside the polymer, which cannot be neutralized by simple contact of the top and bottom surface of polymer film. This ensures long term stability of the sensor performance. Figure 10f shows the results of the pressure–voltage relationship of the sensor. A linear relationship existed when the applied pressure was less than 3 kPa. The coefficient was calculated to be −0.384 V/kPa. The rate of voltage reduction decreased upon further increasing pressure, and, eventually, the voltage reached a stable value. This is because at such pressure and large deformation, the top surface was in direct contact with the bottom surface, preventing any further deflection and charge induction. Nevertheless, the results show that the COC piezoelectric sensor was capable of position specific pressure sensing or pressure mapping.

As primitive as the prototype system is, the 3D printed piezoelectrets have great potential. They require very low power and energy consumption and are suitable for both high rate and low rate force sensing. Moreover, the 3D printing technology presented herein is scalable, and the piezoelectric activity and sensor performance can be easily tailored by the structure parameters. All these factors make them an attractive candidate for a large area-distributed sensing network.

## 4. Conclusions

COC piezoelectrets were fabricated using a 3D printing approach. The approach should provide well controlled structures for tailored electromechanical properties. Using a response surface method, the structure was optimized, and the *d*_33_ of the piezoelectrets with optimal geometry reached ~9000 pC/N, an exceptionally high activity. The charge built up process was further investigated by hysteresis loop, which found that charging followed Paschen breakdown and the critical breakdown voltage can be adequately predicted using a layer model. Furthermore, a physics-based model for $d_{33}$ was developed by combining analytical and finite element analysis, and the model prediction agreed reasonably well with experimental measurements. The 3D printing method presented may be a viable scalable approach to fabricate large area, high-performance pressure sensor arrays with designed sensitivity for pressure mapping, which was demonstrated using a 2 × 2 sensor array.

## Figures and Tables

**Table 1:** Parameter setting in central composite design of the optimization.

Experimental Setup	Pillar Height, d (mm)	Pillar Distance, L (mm)	Pillar Height (Coded Variables)	Pillar Distance (Coded Variables)
1	0.3	15	−1.414	0
2	0.5	12	−1	−1
3	0.5	18	1	1
4	1	11	0	−1.414
5	1	15	0	0
6	1	19	0	1.414
7	1.5	12	1	−1
8	1.5	18	1	1
9	1.8	15	1.414	0

## Data Availability

The data presented in this study are available on request from the corresponding author.

## References

[1] Savolainen A., Kirjavainen K. (1989). Electrothermomechanical Film. Part I. Design and Characteristics. J. Macromol. Sci. Part A Chem..

[2] Bauer S., Gerhard-Multhaupt R., Sessler G.M. (2004). Ferroelectrets: Soft Electroactive Foams for Transducers. Phys. Today.

[3] Wegener M., Bauer S. (2005). Microstorms in Cellular Polymers: A Route to Soft Piezoelectric Transducer Materials with Engineered Macroscopic Dipoles. ChemPhysChem.

[4] Samadi A., Hasanzadeh R., Azdast T., Abdollahi H., Zarintaj P., Saeb M.R. (2020). Piezoelectric Performance of Microcellular Polypropylene Foams Fabricated Using Foam Injection Molding as a Potential Scaffold for Bone Tissue Engineering. J. Macromol. Sci. Part B.

[5] Zhang X., Huang J., Chen J., Wan Z., Wang S., Xia Z. (2007). Piezoelectric properties of irradiation-crosslinked polypropylene ferroelectrets. Appl. Phys. Lett..

[6] Wang H., Li Y., Wang X., Liu Z., Ahmed M.F., Zeng C. (2021). Preparation and Characterization of PiezoelectricI Foams Based on Cyclic Olefin Copolymer. Eng. Sci..

[7] Li Y., Zeng C. (2013). Low-Temperature CO_2_-Assisted Assembly of Cyclic Olefin Copolymer Ferroelectrets of High Piezoelectricity and Thermal Stability. Macromol. Chem. Phys..

[8] Altafim R.P., Rychkov D., Wirges W., Gerhard R., Basso H., Altafim R.C., Melzer M. (2012). Laminated tubular-channel ferroelectret systems from low-density polyethylene films and from fluoroethylene-propylene copolymer films-A comparison. IEEE Trans. Dielectr. Electr. Insul..

[9] Voronina O., Wegener M., Wirges W., Gerhard R., Zirkel L., Münstedt H. (2007). Physical foaming of fluorinated ethylene-propylene (FEP) copolymers in supercritical carbon dioxide: Single-film fluoropolymer piezoelectrets. Appl. Phys. A.

[10] Liu Z., Zeng M., Wang H., Wang X., Li Y., Zeng C. (2022). Toward Flexible Piezoelectrets with High Environmental Stability: A Hybrid Approach. ES Mater. Manuf..

[11] Zhang X., Zhang X., You Q., Sessler G.M. (2013). Low-Cost, Large-Area, Stretchable Piezoelectric Films Based on Irradiation-Crosslinked Poly(propylene). Macromol. Mater. Eng..

[12] Zhang X., Sessler G.M., Wang Y. (2014). Fluoroethylenepropylene ferroelectret films with cross-tunnel structure for piezoelectric transducers and micro energy harvesters. J. Appl. Phys..

[13] Zhang X., Zhang X., Sessler G.M., Gong X. Piezoelectric Performance of Polytetrafluoroethylene Ferroelectrets. Proceedings of the 2013 Annual Report Conference on Electrical Insulation and Dielectric Phenomena.

[14] Wirges W., Wegener M., Voronina O., Zirkel L., Gerhard-Multhaupt R. (2007). Optimized Preparation of Elastically Soft, Highly Piezoelectric, Cellular Ferroelectrets from Nonvoided Poly(ethylene Terephthalate) Films. Adv. Funct. Mater..

[15] NunesPelle P.S., Ohlsson P.D., Ordeig O., Kutter J. (2010). Cyclic olefin polymers: Emerging materials for lab-on-a-chip applications. Microfluid. Nanofluid..

[16] Saarimaki E., Paajanen M., Savijarvi A., Minkkinen H., Wegener M., Voronina O., Schulze R., Wirges W., Gerhard-Multhaupt R. (2006). Novel heat durable electromechanical film: Processing for electromechanical and electret applications. IEEE Trans. Dielectr. Electr. Insul..

[17] Montanari G.C., Fabiani D., Ciani F., Motori A., Paajanen M., Gerhard R., Wegener M. (2007). Charging properties and time-temperature stability of innovative polymeric cellular ferroelectrets. IEEE Trans. Dielectr. Electr. Insul..

[18] Savijarvi A.M., Paajanen M., Saarimaki E., Minkkinen H. Novel Heat Durable Electromechanical Films: Cellular Film Making from Cyclic Olefin Polymers. Proceedings of the 2005 12th International Symposium on Electrets.

[19] Wegener M., Paajanen M., Voronina O., Schulze R., Wirges W., Gerhard-Multhaupt R. Voided Cyclo-Olefin Polymer Films: Ferroelectrets with High Thermal Stability. Proceedings of the 2005 12th International Symposium on Electrets.

[20] Hillenbrand J., Sessler G. (2000). Piezoelectricity in cellular electret films. IEEE Trans. Dielectr. Electr. Insul..

[21] Sessler G.M., Hillenbrand J. (1999). Electromechanical response of cellular electret films. Appl. Phys. Lett..

[22] Lindner M., Bauer-Gogonea S., Bauer S., Paajanen M., Raukola J. (2002). Dielectric barrier microdischarges: Mechanism for the charging of cellular piezoelectric polymers. J. Appl. Phys..

[23] Tuncer E., Wegener M. (2004). Elastic properties of highly anisotropic thin poly(propylene) foams. Mater. Lett..

[24] Qiu X. (2010). Patterned piezo-, pyro-, and ferroelectricity of poled polymer electrets. J. Appl. Phys..

[25] Tuncer E. (2005). Numerical calculations of effective elastic properties of two cellular structures. J. Phys. D Appl. Phys..

[26] Joseph V.R., Yan H. (2015). Engineering-Driven Statistical Adjustment and Calibration. Technometrics.

[27] Joseph V.R., Melkote S.N. (2009). Statistical Adjustments to Engineering Models. J. Qual. Technol..

[28] Montgomery D.C. (2008). Design and Analysis of Experiments.

[29] Arockiarajan A., Delibas B., Menzel A., Seemann W. (2005). Studies on nonlinear electromechanical behavior of piezoelectric materials using finite element modeling. Continuity.

[30] Yeh T.-J., Lu S.-W., Wu T.-Y. (2005). Modeling and Identification of Hysteresis in Piezoelectric Actuators. J. Dyn. Syst. Meas. Control.

[31] Ding C., Cao J., Chen Y. (2019). Fractional-order model and experimental verification for broadband hysteresis in piezoelectric actuators. Nonlinear Dyn..

